# P2X7 Receptor Stimulation Is Not Required for Oxalate Crystal-Induced Kidney Injury

**DOI:** 10.1038/s41598-019-56560-2

**Published:** 2019-12-27

**Authors:** Hannah L. Luz, Martin Reichel, Robert J. Unwin, Kerim Mutig, Ana C. Najenson, Louise M. Tonner, Kai-Uwe Eckardt, Frederick W. K. Tam, Felix Knauf

**Affiliations:** 1 Department of Nephrology and Medical Intensive Care, Charité – Universitätsmedizin Berlin, Berlin, Germany; 2 Friedrich-Alexander-Universität Erlangen-Nürnberg (FAU), Erlangen, Germany; 3 Centre for Nephrology, Royal Free Hospital, University College London, London, UK; 4 Department of Vegetative Anatomy, Charité – Universitätsmedizin Berlin, Berlin, Germany; 5 Department of Pharmacology, I.M. Sechenov First Moscow State Medical University (Sechenov University), Moscow, Russian Federation; 6 Centre of inflammatory disease, Department of Medicine, Hammersmith Hospital, Imperial College London, London, UK

**Keywords:** End-stage renal disease, Inflammasome

## Abstract

Oxalate crystal-induced renal inflammation is associated with progressive kidney failure due to activation of the NLRP3/CASP-1 inflammasome. It has been suggested previously that purinergic P2X7 receptor signaling is critical for crystal-induced inflammasome activation and renal injury. Therefore, we investigated the role of the P2X7 receptor in response to crystal-induced cytokine release, inflammation, and kidney failure using *in vitro* and *in vivo* models. Dendritic cells and macrophages derived from murine bone marrow and human peripheral blood mononucleated cells stimulated with calcium-oxalate crystals, monosodium urate crystals, or ATP lead to the robust release of interleukin-1beta (IL-1ß). Treatment with the P2X7 inhibitor A740003 or the depletion of ATP by apyrase selectively abrogated ATP-induced, but not oxalate and urate crystal-induced IL-1ß release. In line with this finding, dendritic cells derived from bone marrow (BMDCs) from *P2X7*^−/−^ mice released reduced amounts of IL-1ß following stimulation with ATP, while oxalate and urate crystal-induced IL-1ß release was unaffected. In sharp contrast, BMDCs from *Casp1*^−/−^ mice exhibited reduced IL-1ß release following either of the three stimulants. In addition, *P2X7*^−/−^ mice demonstrated similar degrees of crystal deposition, tubular damage and inflammation when compared with WT mice. In line with these findings, increases in plasma creatinine were no different between WT and *P2X7*^−/−^ mice. In contrast to previous reports, our results indicate that P2X7 receptor is not required for crystal-induced CKD and it is unlikely to be a suitable therapeutic target for crystal-induced progressive kidney disease.

## Introduction

The kidney is highly predisposed to crystalopathies, since one of its main functions is to filter, secrete and concentrate substances via urine formation. Oxalic acid, which is not significantly metabolized by mammals^1,2^, is mainly excreted by the kidney^1–3^. Oxalate, its ionized form, can form highly insoluble complexes with calcium^2^. When oxalate homeostasis is disturbed, either by endogenous over-production (primary hyperoxaluria)^1,4^, excessive exogenous provision (secondary hyperoxaluria)^1,2,4^, or renal dysfunction, oxalate accumulates in the body, may harm the kidneys and lead to end-stage renal disease^4^. Inflammasomes are large multiprotein complexes activated in response to pathogen- and damage-associated molecular patterns (PAMPs, DAMPs)^5,6^. Various inflammasomes have been identified, but by far the best studied is the Nacht Domain-, Leucin-Rich Repeat-, and PYD containing Protein 3 (NLRP3, Nalp3, Crypopyrin) inflammasome, mainly expressed in myeloid cells such as dendritic cells and macrophages. Several crystalline materials, among them oxalate^7,8^, urate^9,10^, cholesterol^11^, silica^12–14^, alum^15^ and hydroxyapatite^16^, have been shown to be capable of activating the inflammasome machinery. Oxalate crystals have been demonstrated to stimulate IL-1ß in dendritic cells and *Nlrp3*^−/−^ mice are protected from oxalate nephropathy^7,8^. Moreover, it is well established that the purinergic receptor P2X7 and ATP are involved in inflammation and immunity. P2X7 is expressed by virtually all cells of innate immunity and mediates NLRP3 inflammasome activation, resulting in IL-1ß release^4,10,12,17^. Moreover, it has been demonstrated that (1) silica^12,18^ and uric acid^10,17^ activate the inflammasome via P2X7 signaling, (2) treatment with a P2X7 inhibitor reduces IL-1ß release^10,12,18^, (3) oxalate crystal-induced ATP release contributes to kidney inflammation^8^ and (4) several studies have suggested that P2X7 may be a suitable pharmacological target in various renal diseases, such as diabetic nephropathy^19,20^, glomerulonephritis^21^, hypertension^20^, kidney injury induced by metabolic syndrome^22^ and ischemic acute kidney injury^23^. Hence, the present study is directed at defining the role of P2X7 receptor in crystal-induced inflammation and kidney disease.

## Results

### Oxalate, urate crystals, and ATP induce IL-1ß release *in vitro*

**Figure 1:**
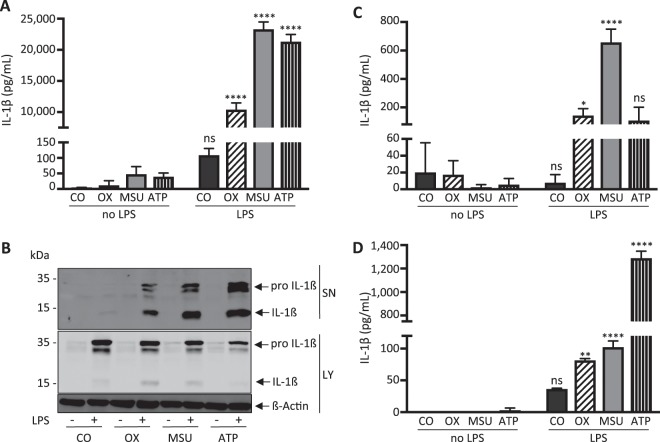
Calcium oxalate, monosodium urate and ATP induce IL-1β release in LPS-primed murine BMDCs, BMDMs and human PBMCs *in vitro*. (**A**,**B**) Murine bone marrow-derived dendritic cells (BMDCs), (**C**) murine bone marrow-derived macrophages and (**D**) human peripheral blood mononuclear cells were primed with LPS (100 ng/ml) for 3 hours before either calcium oxalate crystals (100 µg/ml) (OX), monosodium urate crystals (300 µg/ml) (MSU) or ATP (5 mM) or no further stimulus as control (CO) were added for an additional 6 hours. Culture supernatants (SN) were collected and concentrations of IL-1ß were measured using ELISA. (**A**,**C**,**D**) In addition, supernatants and whole cell lysates (LY) from stimulated murine BMDCs were analyzed by western blotting. (**B**) All three stimuli induced IL-1ß release in all three cell types. Data are presented as mean ± SD of a representative experiment of a total of nine each performed with triplicate biological samples. Statistical analysis was performed using two-way ANOVA and post hoc analysis. ****P < 0.0001; **P < 0.01; *P < 0.05; ns, not significant compared with control treatment (without LPS). To improve the clarity of the presented western blots, blots are displayed in a cropped version. Full-length gels are presented in Supplementary Fig. 1.

In a first series of experiments we investigated IL-1ß cytokine release from BMDCs in response to calcium oxalate crystals, monosodium urate crystals and ATP *in vitro*. As shown in Fig. 1A,B, all three stimuli activated murine BMDCs to synthesize (lysate) and release (supernatant) IL-1β following priming with LPS as previously reported^8,10,24^. To examine whether our findings are specific to BMDCs or can be elicited in different mononuclear cells, we further investigated BMDMs. As demonstrated in Fig. 1C, BMDMs released IL-1ß in response to crystals and ATP similar to BMDCs. In order to demonstrate that our findings are not limited to murine mononuclear cells, we next prepared human PBMCs and demonstrated the release of IL-1ß in response to all three stimuli as shown in Fig. 1D.

### Crystal-induced IL-1ß secretion is independent of P2X7 *in vitro*

**Figure 2:**
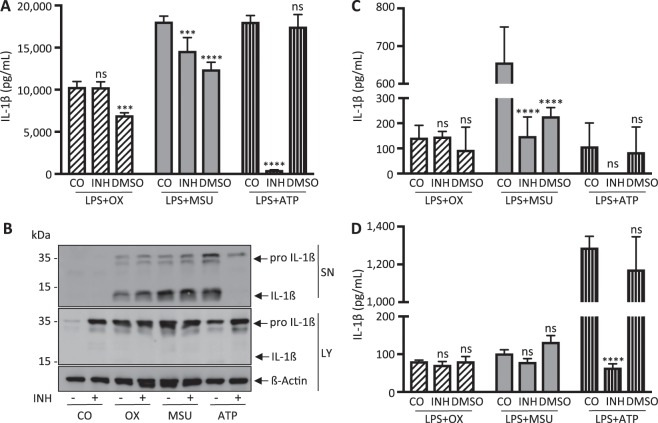
Pharmacological inhibition of P2X7 does not affect crystal-induced IL-1β release *in vitro*. The P2X7 inhibitor A740003 (100 µM) (INH) dissolved in DMSO was applied to LPS-primed (**A**,**B**) murine bone marrow-derived dendritic cells, (**C**) murine bone marrow-derived macrophages and (**D**) human peripheral blood mononuclear cells. The inhibitor was added to the cells 15 minutes before stimulation with ATP or crystals for an additional 6 hours. A740003 did not affect oxalate (100 µg/ml) nor urate (300 µg/ml) crystal induced IL-1ß release. In contrast, ATP-induced IL-1ß release was completely abrogated in all cell types. IL-1ß release was analyzed using ELISA (**A**,**C**,**D**) for cell-free supernatants and western blotting (**B**) for whole cell lysates and supernatants from murine BMDCs. Note that DMSO affected IL-1ß release! Data are presented as mean ± SD of a representative experiment of a total of four each performed with triplicate biological samples. Statistical analysis was performed using two-way ANOVA and post hoc analysis. ****P < 0.0001; ***P < 0.001; ns, not significant compared with control treatment. To improve the clarity of the presented western blots, blots are displayed in a cropped version. Full-length gels are presented in Supplementary Fig. 2.

To investigate a potential role of P2X7 receptor in crystal-induced NLRP3-Casp1-inflammasome activation and cytokine release, we pretreated BMDCs, BMDMs and PBMCs with the selective P2X7 receptor antagonist A740003. As shown in Fig. 2A–D, pharmacological inhibition of P2X7 selectively abrogated ATP-induced IL-1ß release into the supernatant (Fig. 2B). In sharp contrast, crystal-induced IL-1ß release remained unaffected. Of note, DMSO applied as a vehicle control resulted in reduced IL-1ß release. This observation is consistent with previous reports that have demonstrated that DMSO downregulates and restrains NLRP3 activation through blockage of mitochondrial ROS generation^25,26^.

**Figure 3:**
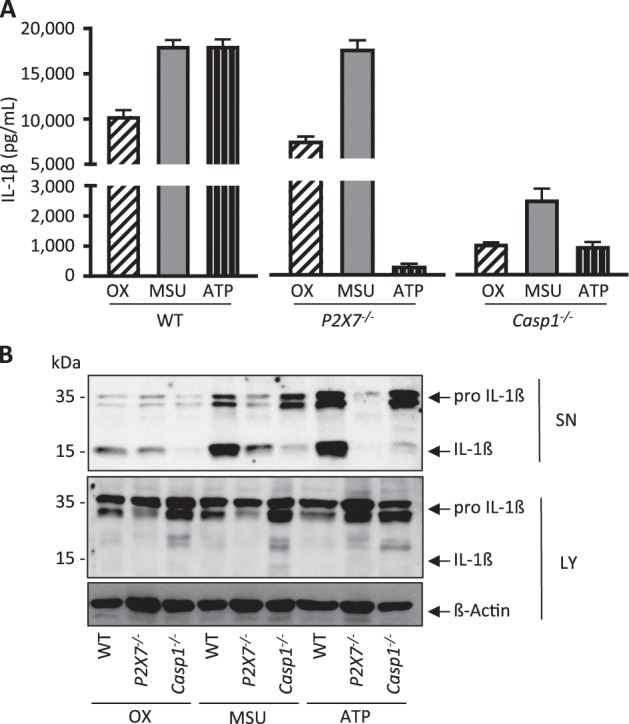
IL-1β secretion is caspase-1 dependent but does not require P2X7 signaling *in vitro*. Murine bone marrow-derived dendritic cells (BMDCs) from WT, *P2X7*^−/−^ and *Casp1*^−/−^ mice were treated with calcium oxalate crystals (100 µg/ml), monosodium urate crystals (300 µg/ml) or ATP (5 mM). (A,B) BMDCs from *P2X7*^−/−^ mice showed completely abrogated ATP-induced IL-1β release, yet crystal-induced cytokine release remained unaffected. In contrast, BMDCs from *Casp1*^−/−^ mice showed reduced IL-1β release for all three stimuli. IL-1ß release was analyzed using (**A**) ELISA and (**B**) western blotting. Data are presented as mean ± SD of a representative experiment of a total of three each performed with triplicate biological samples. To improve the clarity of the presented western blots, blots are displayed in a cropped version. Full-length gels are presented in Supplementary Fig. 3.

In a next series of experiments, we examined BMDCs from P2X7 deficient mice. As shown in Fig. 3A, crystal-induced IL-1ß release remained unaffected in BMDCs from *P2X7*^−/−^ mice, whereas ATP-induced IL-1ß was completely abrogated. In sharp contrast, BMDCs from *Casp1*^−/−^ mice exhibited significantly (P < 0.05) reduced IL-1ß release following either of the three stimulants, supporting the hypothesis of a defective final common pathway for both crystals and ATP in *Casp1*^−/−^ mice.

### Crystal-induced IL-1ß secretion is independent of purinergic signalling *in vitro*

**Figure 4:**
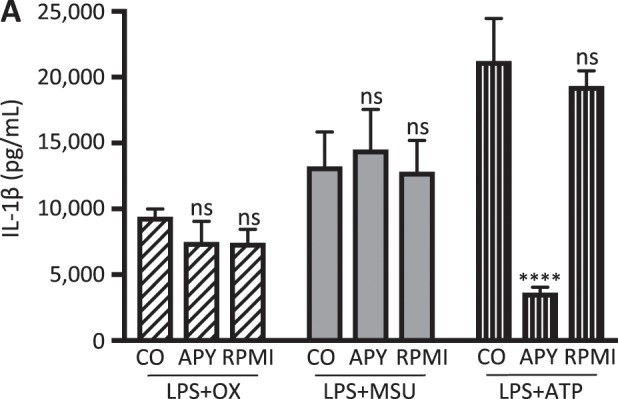
Pharmacological hydrolysis of ATP abrogates ATP-induced IL-1β secretion but does not affect crystal-induced IL-1ß secretion *in vitro*. Murine bone marrow-derived dendritic cells were treated with apyrase (10 U/ml) (APY) 15 minutes before adding either oxalate or urate crystals or ATP to the cells to induce hydrolysis of ATP. Crystal induced IL-1ß release remained unaffected whereas ATP-induced IL-1ß release was abrogated. Data are presented as mean ± SD of a representative experiment of a total of three each performed with triplicate biological samples. Statistical analysis was performed using two-way ANOVA and post hoc analysis. ****P < 0.0001; ns, not significant compared with control treatment.

Since it has also been suggested that other P2X receptors such as P2X4 could play a role in inflammasome activation and IL-1ß cytokine release^24^, we next examined the effect of depleting ATP on crystal-induced cytokine release. As shown in Fig. 4, BMDCs treated with apyrase, an enzyme catalyzing ATP hydrolysis, completely abrogated ATP-induced IL-1ß release. In contrast, crystal-induced IL-1ß release remained unaffected, arguing against a significant involvement of purinergic signaling in crystal-induced IL-1ß release.

### *P2X7*^−/−^ mice are not protected from crystal-induced renal failure *in vivo*

**Figure 5:**
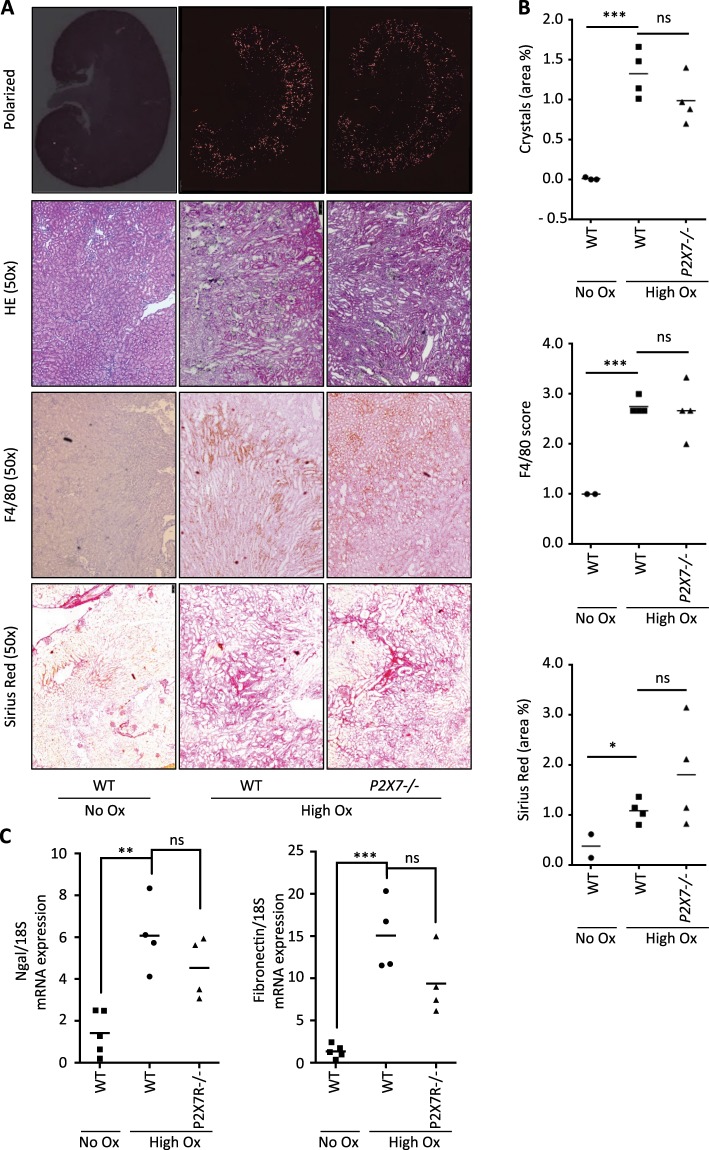
*P2X7*^−/−^ mice demonstrate similar amounts of renal crystal deposition and inflammation *in vivo*. WT and *P2X7*^−/−^ mice were placed on a high soluble oxalate diet to induce renal failure. A separate group of WT mice received a control diet. (**A**,**B**) Whole kidney scans show no significant difference in crystal deposition, tubular damage, inflammation and renal fibrosis between WT and *P2X7*^−/−^ mice on high oxalate diet. (**C**) mRNA levels of renal injury and fibrosis markers Ngal and fibronectin showing no significant difference between WT and *P2X7*^−/−^ mice on high oxalate diet. Data are presented as mean. n = 3–4 animals. Statistical analysis for the histopathological evaluation (**B**) was performed using unpaired t-test. Statistical analysis for mRNA expression (**C**) was performed using one-way ANOVA. ***P < 0.001; **P < 0.01; ns, not significant compared with mice on control diet (0% calcium/0% oxalate).

**Figure 6:**
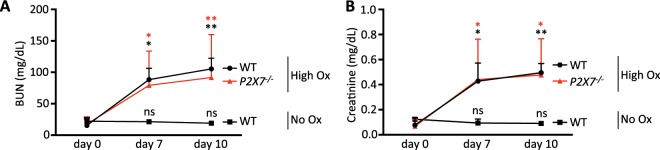
*P2X7*^−/−^ mice are not protected from oxalate-induced renal failure *in vivo*. (**A**,**B**) Renal function was assessed measuring plasma creatinine and blood urea nitrogen (BUN) from retro-orbital blood samples. *P2X7*^−/−^ mice showed no protection from oxalate nephropathy *in vivo*. Statistical analysis was performed using two-way ANOVA. **P < 0.01; *P < 0.05; ns, not significant compared with mice on control diet (0% calcium/0% oxalate).

It has previously been suggested that ATP released from necrotic tubular cells may trigger NLRP3 inflammasome activation *in vivo*^8^. Moreover, treatment with apyrase has been shown to mitigate oxalate crystal-induced kidney injury^8^. Therefore, in a next series of experiments we examined the potential role of P2X7 receptor in crystal-induced progressive kidney injury *in vivo*. Crystal-induced renal failure was induced by feeding mice a high soluble oxalate diet for 10 days as previously described^7^. Control animals were fed an oxalate-free diet. As shown in Fig. 5A, renal histology of wild type and *P2X7*^−/−^ mice fed a high soluble oxalate diet demonstrated profound crystal deposition, and analysis of hematoxylin and eosin (HE) staining revealed a high degree of tubular damage, as indicated by dilatation and rupture of tubules (Fig. 5A). This finding was also reflected in elevated mRNA expression of the renal injury biomarker, neutrophil gelatinase-associated lipocalin (NGAL)^27^, and the fibrosis marker, fibronectin (Fig. 5C). In addition, severe inflammation and fibrosis was noted as measured by infiltration of inflammatory cells positive for F4/80 and Sirius red staining (Fig. 5A). There was no difference in severity of crystal deposition, inflammation and fibrosis between WT and *P2X7*^−/−^ mice with oxalate nephropathy (Fig. 5B). We next examined the renal function of *P2X7*^−/−^ compared to wild type mice. As shown in Fig. 6, baseline blood urea nitrogen (BUN) and creatinine were determined on a control diet (oxalate-free diet). Subsequently, the diet was switched to a high soluble oxalate diet and progression of renal failure was monitored longitudinally at 7 and 10 days. Wild type and *P2X7*^−/−^ mice demonstrated similar degrees of progressive renal failure over the following 10-day period as indicated by rising plasma BUN and creatinine compared to mice receiving a control diet suggesting that P2X7 receptor signaling is not involved in oxalate-crystal induced progressive renal failure. To exclude that our finding is due to a different strain of wild type mice used as compared with *P2X7*^−/−^ mice, we repeated our experiments with wild-type littermates from our *P2X7*^−/−^ strain. As shown in Supplementary Figs. 5 and 6, *P2X7*^−/−^ mice and their wild type littermates receiving a soluble oxalate diet again demonstrate progressive kidney failure when compared with mice receiving a control diet (oxalate-free diet). mRNA expression of the renal injury biomarker, neutrophil gelatinase-associated lipocalin (NGAL)^27^, and the fibrosis marker, fibronectin (Supplementary Fig. 6) again demonstrated no difference between WT littermates and *P2X7*^−/−^ mice, arguing against a strain effect to account for our findings.

## Discussion

Purinergic receptors have received major attention as drug targets in renal disease because of their role in glomerular, tubular, and vascular cell damage^19,20,23^. However, several P2X7 receptor antagonists have completed phase 2 clinical trials and these compounds have failed to deliver the expected benefit and interest in P2X7 receptor has declined^28^. Since CKD is a ‘catch-all’ term and syndrome representing a mix of various distinct disease etiologies, it is necessary to define the underlying pathophysiology and select appropriate patient cohorts to test any novel therapeutic pharmacological approach. To date, the involvement of P2X7 receptor in crystal-induced IL-1ß release and inflammasome activation is not clear and indeed rather contradictory. Previous reports have demonstrated that uric acid^17^ or silica crystals lead to ATP and IL-1ß release in BMDMs^29^. Similarly, crystal-induced lung injury was reduced in *P2X7*^−/−^ mice as compared with WT mice^30^. *In vitro* studies using specific pharmacological inhibitors demonstrated that the P2X7 receptor participates in crystal-induced IL-1ß release, reactive oxygen production and particle phagocytosis^18,30^. However, several groups of investigators have failed to confirm a role for P2X7 receptor in crystal-induced inflammasome activation and IL-1 release using BMDCs from *P2X7*^−/−^ mice^13,16,31,32^. Specifically, membrane permeation by crystalline materials was not dependent on P2X7 receptors and was suggested to be secondary to phagocytosis, because it was strongly inhibited by cytochalasin B and latrunculin^32^. Our findings using crystals such as monosodium urate and oxalate are in line with the latter observation.

The contrasting findings may be explained by different methodological approaches of preparing cell types, priming of cells or use of different sizes of crystals. Of interest, P2X7 inhibitors such as A740003 are dissolved in DMSO, which is a potent inflammasome inhibitor^25^ that may have been overlooked if this vehicle was not examined separately. Moreover, our data suggest that even in the setting of a complex interaction of tubular cell damage and inflammatory cell activation *in vivo*, P2X7 receptor signaling is dispensable. Using a model of acute oxalate crystal-induced kidney injury, it has been suggested that depletion of ATP by apyrase^8^ may prevent kidney failure. However, our findings suggest that this is unlikely to be mediated by the P2X7 receptor, because we observed no protection of crystal-induced renal inflammation or organ failure in *P2X7*^−/−^ mice when compared with WT mice. Nevertheless, we cannot exclude the *in vivo* participation of other purinergic signaling pathways.

Together, our current findings suggest that while NLRP3 deficiency or its pharmacological inhibition prevents renal inflammation and failure^7,8,33^, P2X7 receptor stimulation is not required for oxalate crystal-induced kidney injury. Therefore, clinical studies examining P2X7 antagonists should not include crystal nephropathies, since this may obscure a potential benefit of these compounds in certain subsets of renal disease.

## Methods

### *In vitro* studies

#### Murine bone marrow-derived dendritic cells and macrophages

Bone marrow-derived dendritic cells (BMDCs) were isolated as previously described^34^ from either C57BL/6N, *P2X7*^−/−^ and *Casp1*^−/−^ mice. In brief, bone marrow cells were isolated from murine femur and tibia and differentiated at 37 °C in RPMI 1640 medium (Thermo Fisher Scientific, Waltham, Massachusetts, USA) supplemented with 10% heat inactivated fetal bovine serum (FBS) (FBS 10270, Gibco®, Thermo Fisher Scientific, Waltham, Massachusetts, USA), 1% penicilline-streptomycine (10,000U/10,000 μg ml^−1^) (Biochrom, Berlin, Germany) and 50 mM 2-Mercaptoethanol (Sigma Aldrich, St. Louis, Missouri, USA) and added recombinant murine granulocyte/monocyte-culture stimulating factor (GM-CSF) (PeproTech, Rocky Hill, New Jersey, USA) at a density of 2.5 × 10^6^ cells 10 ml^−1^ for 8 days. Fresh medium and GM-CSF were added on day 3 and 6 after isolation. Cells were harvested on day 8 and viable cells were determined using 0.4% trypan blue solution (Thermo Fisher Scientific, Waltham, Massachusetts, USA). For experiments, cells were seeded into multi-well tissue culture plates at a density of 1 × 10^6^ cells ml^−1^. For bone marrow-derived macrophages (BMDMs), bone marrow cells were obtained from murine femur and tibia and cultured in RPMI 1640 medium supplemented with 20% FBS, 30% conditioned media from L929 cells (containing macrophage-colony stimulating factor), 25 mM HEPES (Carl Roth, Karlsruhe, Germany) and 1% penicillin/streptomycin at 37 °C as described previously^35,36^. Cells were differentiated for 7 days and fresh media was added on days 4 and 6 of culturing. BMDMs were seeded for stimulation in 24-well tissue culture plates at a density of 200,000 cells ml^−1^.

#### Human peripheral blood mononuclear cells

Peripheral blood mononuclear cells (PBMCs) were isolated from healthy voluntary donors as described previously. Briefly, 15 ml of Lymphoflot (BioRad, Hercules, California, USA) were added to 20 ml of EDTA-blood and centrifuged 25 minutes at 2,000 rpm (Heraus Megafuge 40 R, Thermo Fisher Scientific, Waltham, Massachusetts, USA) and 20 °C without breaks and without acceleration. PBMCs were obtained in 10 ml PBS (Biochrom, Berlin, Germany) and washed three times. Washed cells were taken up in RPMI 1640 medium supplemented with 10% FBS, 1% penicilline-streptomycine and 50 mM 2-Mercaptoethanol. Cells were seeded for stimulation at a density of 2 × 10^6^ cells ml^−1^ in 24-well tissue plates.

#### Cell culturing

Cells were stimulated with 100 ng ml^−1^ LPS for 3 hours (Ultra pure lipopolysaccharide from E. coli 0111:B4 strain, InvivoGen, San Diego, California, USA), followed by addition of a second stimulus for supplemental 6 hours. The second stimulus consisted either of calcium oxalate (100 μg ml^−1^ from a 1 mg ml^−1^ stock stored at 4 °C) (Sigma Aldrich, St. Louis, Montana, USA), ATP (5 mM from a prepared 100 mM stock solution stored at −20 °C) (InvivoGen, San Diego, California, USA) or monosodium urate crystals (300 μg ml^−1^ from 5 mg ml^−1^ stock solution stored at −20 °C) (InvivoGen, San Diego, California, USA). The selective P2X7 inhibitors A740003 or apyrase (both from Sigma Aldrich, St. Louis, Montana, USA) were applied 15 minutes prior to the addition of the second stimulus. A740003 was dissolved in pure DMSO and cells were treated with 100 μM A740003^10^ or the equivalent amount of DMSO alone (0.5%). Apyrase was dissolved in RPMI and cells were treated with 10U apyrase ml^−1^ or the corresponding amount of RPMI only. At the end of every series supernatants were collected and RIPA buffer (Sigma Aldrich, St. Louis, Montana, USA) containing complete EDTA-free protease inhibitor (Roche, Mannheim, Germany) was added to each well to gain whole cell lysates.

#### Western blot

Cells lysated with RIPA buffer containing protease inhibitor as described above were sonicated using a Bioruptor Plus (Diagenode, Philadelphia, Pennsyvania, USA). Protein concentration was determined using the standard Lowry method using the Bio-Rad DC™ Protein Assay (Bio-Rad, Hercules, California, USA). Cell-free supernatants (500 μl) were concentrated with ultra filters (Vivaspin 500, Membrane 5,000 MWCO PES, Sartorius Stedim Biotech, Göttingen, Germany). 20 µl of concentrated supernatants or 20 μg of cell lysates were subjected to 12% SDS-PAGE and immunoblotted with IL-1β antibodies 1:1000 (Sigma Aldrich, St. Louis, Missouri, USA). Peroxidase-conjugated monoclonal antibody against ß-actin (Sigma Aldrich, St. Louis, Missouri, USA) was used as a loading control.

#### Cytokine enzyme-linked immunosorbant assay (ELISA)

Secreted levels of IL-1ß were measured from supernatant using BD OptEIA^TM^ ELISA Set (BD Biosciences, Franklin Lakes, New Jersey, USA) according to manufacturer’s instructions.

### *In vivo* studies

#### Animal studies

All experiments were performed on male age- and gender-matched 8–12 week old mice. C57BL/6 N mice (wild type control animals) were purchased from Charles River Laboratories (Sulzfeld, Germany). *P2X7*^−/−^ (B6-P2rx7^tm1Ipch^) were a gift  from GlaxoSmithKline and have been described in detail elsewhere^37^. The absence of mRNA transcript was confirmed using qPCR as shown in Supplementary Fig. 4. *Casp1*^−/−^ (B6-Casp1^tm2.1Flv^)^38^ were kindly provided by Till Strowig (Helmholtz Centre for Infection Research, Braunschweig, Germany). The mice were housed in groups of four with a 12-hour dark/light cycle with unlimited access to food and water. Mouse synthetic diets were obtained from Ssniff (Ssniff-Spezialdiäten GmbH, Soest, Germany). The high soluble oxalate diet was manufactured by adding 50 mmol sodium oxalate kg^−1^ to a virtually calcium- and oxalate free diet as previously described^39^. All mice were fed with a calcium- and oxalate free diet three days prior to switching to the high-oxalate diet. All experimental protocols were approved by the Committee on Animal Health and Care of the Government of Unterfranken (Permit Number: 55.2-2532.1-40/14) and conform to international guidelines on the ethical use of animals.

#### Assessment of renal function

Kidney function was monitored by determination of blood urea nitrogen (BUN) and plasma creatinine. Retro-orbital blood samples were collected at indicated time points as previously described^7^. Plasma BUN and creatinine levels were measured using a Cobas Integra 800 auto-analyzer (Roche, Germany).

#### Histopathological evaluation

Kidney sections from C57BL/6N and *P2X7*^−/−^ mice were fixed in zinc (in TRIS-based buffer) over night, embedded in paraffin, and stained with hematoxylin and eosin (HE). Whole kidney sections were scanned with polarization microscopy using a Leica microscope (Leica DM 6000B, Wetzlar, Germany). Oxalate crystal deposition was quantified using ImageJ software (National Institutes of Health, Bethesda, Maryland, USA). By setting an intensity threshold crystals were separated from background tissue. Total pixels above this threshold are expressed as a percentage of total kidney surface area as previously described^7^. Tubulointerstitial fibrosis was detected by Sirius Red staining. Kidney sections were stained with 0.1% Sirius Red in saturated picric acid for 1 hour, followed by dehydration with 100% ethanol and finally washed in xylene. Sirius red positive areas were detected in whole kidney scans using ImageJ software as previously described^40^ and are presented as percentage area per kidney scan.

#### Immunostaining

2 μm sections of murine kidneys fixed in 4% paraformaldehyde were used for immunostaining as previously described^7^. Briefly, an avidin-biotin immunoperoxidase method was used (ABC-Kit, Vector laboratories, Burlingame, CA, USA) in combination with ImmPACT DAB as substrate (Vector laboratories, Burlingame, CA, USA) and monoclonal rat anti mouse F4/80 (1:500, BioRad, Hercules, California, USA) antibodies directed against macrophages/monocytes. Peroxidase positive areas (dark staining) were quantified in whole kidney scans by three different observers in blinded fashion using a five-point scoring system as following: 1, none; 2, <25%; 3, 25%-50%; 4, 51%-75%; 5, >75%.

#### Real-time reverse transcription-polymerase chain reaction (RT-PCR)

Total RNA was isolated from frozen kidney tissue using PureLink RNA Mini Kit (Ambion life technologies, California, USA) following manufacturer’s instructions, adding treatment with DNase (Qiagen, Venlo, Netherlands). Frozen tissue was homogenized in 600 μl RNA lysis buffer containing 1% tris(2-carboxyethyl)phosphine (Marchery-Nagel, Düren, Germany) using a T25 basic ULTRA-TURRAX® dispersing device (IKA-Werke GmbH & CO. KG, Staufen, Germany). RNA quantity was assessed spectrophotometrically using the Nanodrop 2000 (Thermo Fisher Scientific, Waltham, Massachusetts, USA). 100 ng of RNA were transcribed into cDNA. All reagents for cDNA preparation including RevertAid Reverse Transcriptase, reaction buffer, RiboLock RNase inhibitor, random hexamer primer and dNTP mix were obtained from Thermo Fisher Scientific (Waltham, Massachusetts, USA). Real-time PCR on cDNA was performed using a StepOne Plus^TM^ Real Time-PCR system (Applied Biosystems, Waltham, Massachusetts, USA) using SYBR Green Master Mix (Thermo Fisher Scientific, Waltham, Massachusetts, USA). All primers were obtained from Sigma Aldrich (St. Louis, Missouri, USA). Primer sequences used are listed in Table 1. mRNA expression values of all genes were normalized to 18S rRNA as a housekeeping gene. For ease of comparison, expression of each gene in wild type animals was set to 1 and values are given relative to their respective control.

**Table 1:** Primer sequences used to determine mRNA expression levels.

Target	Primer sequence (5′-3′)
18S	Forward: TTGATTAAGTCCCTGCCCTTTGT Reverse: CGATCCGAGGGCCTCACTA
Fibronectin	Forward: GTGTAGCACAACTTCCAATTACGAA Reverse: GGAATTTCCGCCTCGAGTCT
NGAL	Forward: GGCAGCTTTACGATGTACAGCAC Reverse: TCTGATCCAGTAGCGACAGCC
P2X7	Forward: CTGGTTTTCGGCACTGGA Reverse: CCAAAGTAGGACAGGGTGGA

#### Statistical analysis

Statistical analysis was performed using unpaired t-test, one- or two-way ANOVA and post-hoc analysis as indicated using GraphPad Prism Version 7.00 (GraphPad Software, La Jolla, CA, USA) assuming normal distribution of the values and equality of variances. For the comparison of two independent groups unpaired t-test was used. One- and two-way ANOVA were used to compare more than two groups and to assess more than one dependent variable, respectively.

## Supplementary information

Supplementary Dataset 1

## Data Availability

The datasets generated and analyzed during the current study are available from the corresponding author at reasonable request.
