# Supplementary material for: P2X7 Receptor Stimulation Is Not Required for Oxalate Crystal-Induced Kidney Injury
Source: Sci Rep. 2019 Dec 27;9:20086. doi: 10.1038/s41598-019-56560-2 (PMC6934555; doi:10.1038/s41598-019-56560-2)
Supplement: Supplementary file 1 — Supplementary Dataset 1 [file 41598_2019_56560_MOESM1_ESM.pdf]

P2X7 Receptor Stimulation Is Not Required for Oxalate Crystal-Induced Kidney Injury

Hannah L. Luz, Martin Reichel, Robert J. Unwin, Kerim Mutig, Ana C. Najenson, Louise M. Tonner, Kai-Uwe Eckardt, Frederick W.K. Tam, Felix Knauf

1 – Uncropped original western blots figure 1B.

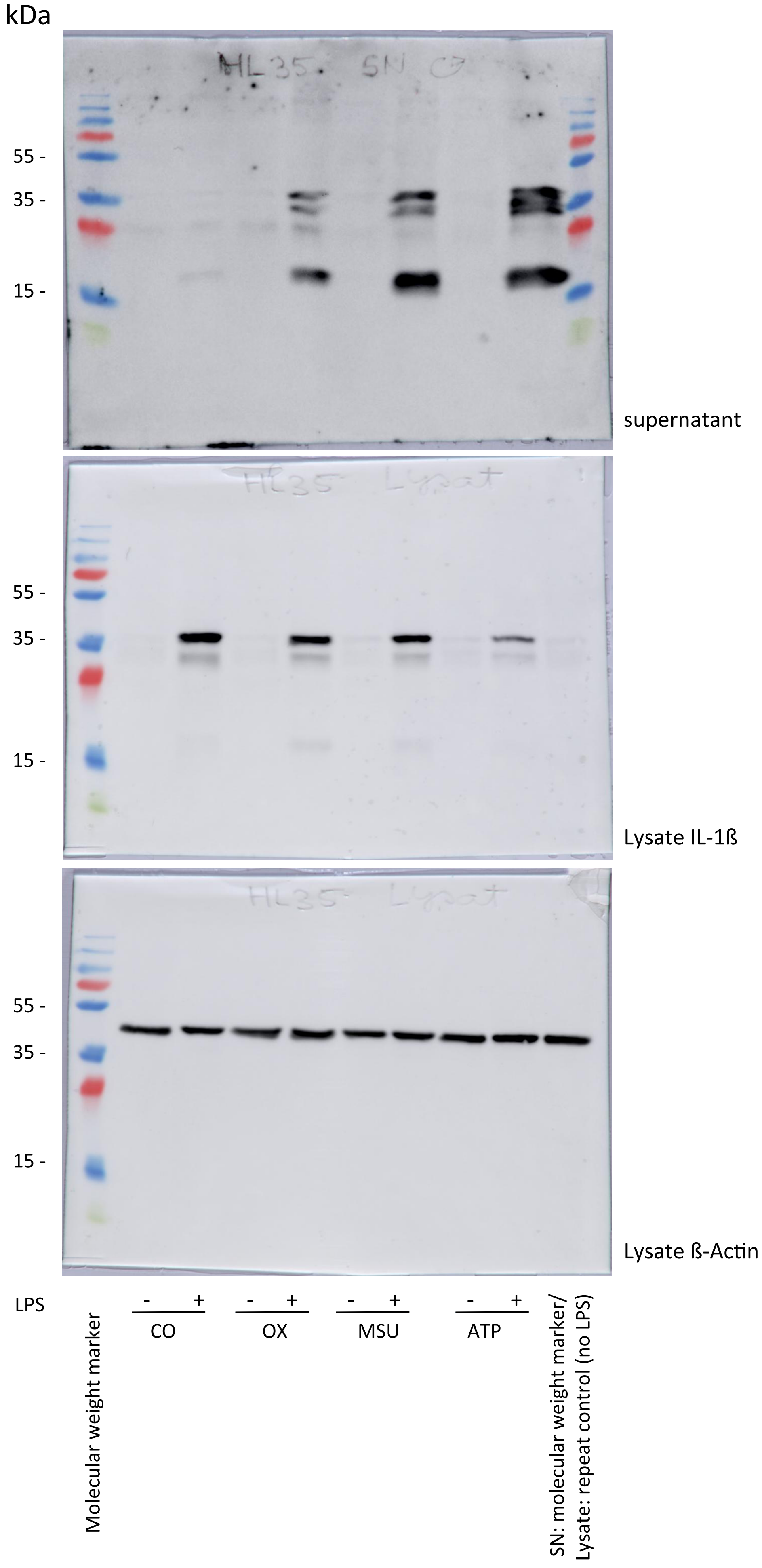

2 – Uncropped original western blots figure 2B.

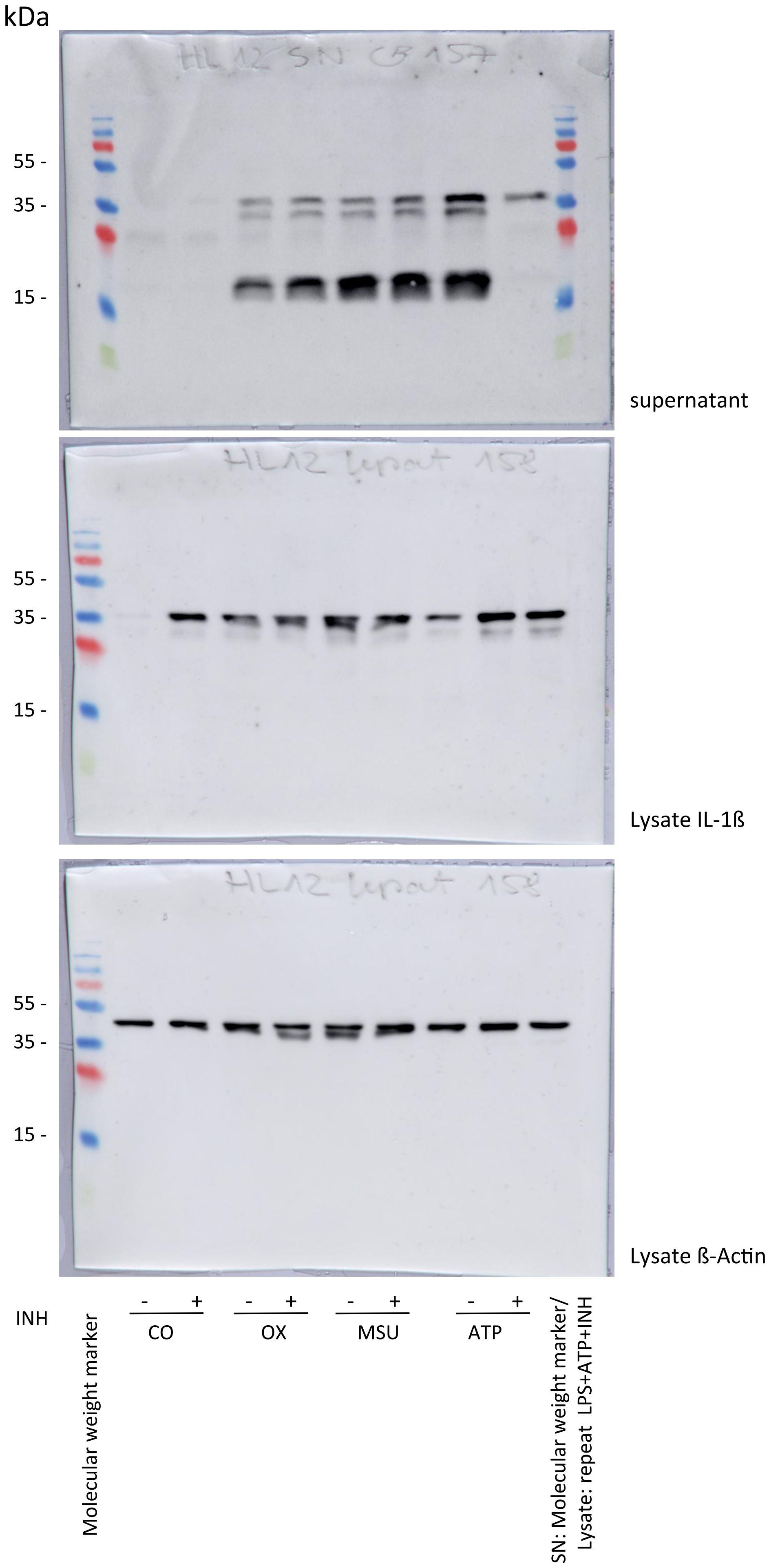

3 – Uncropped original western blots figure 3B.

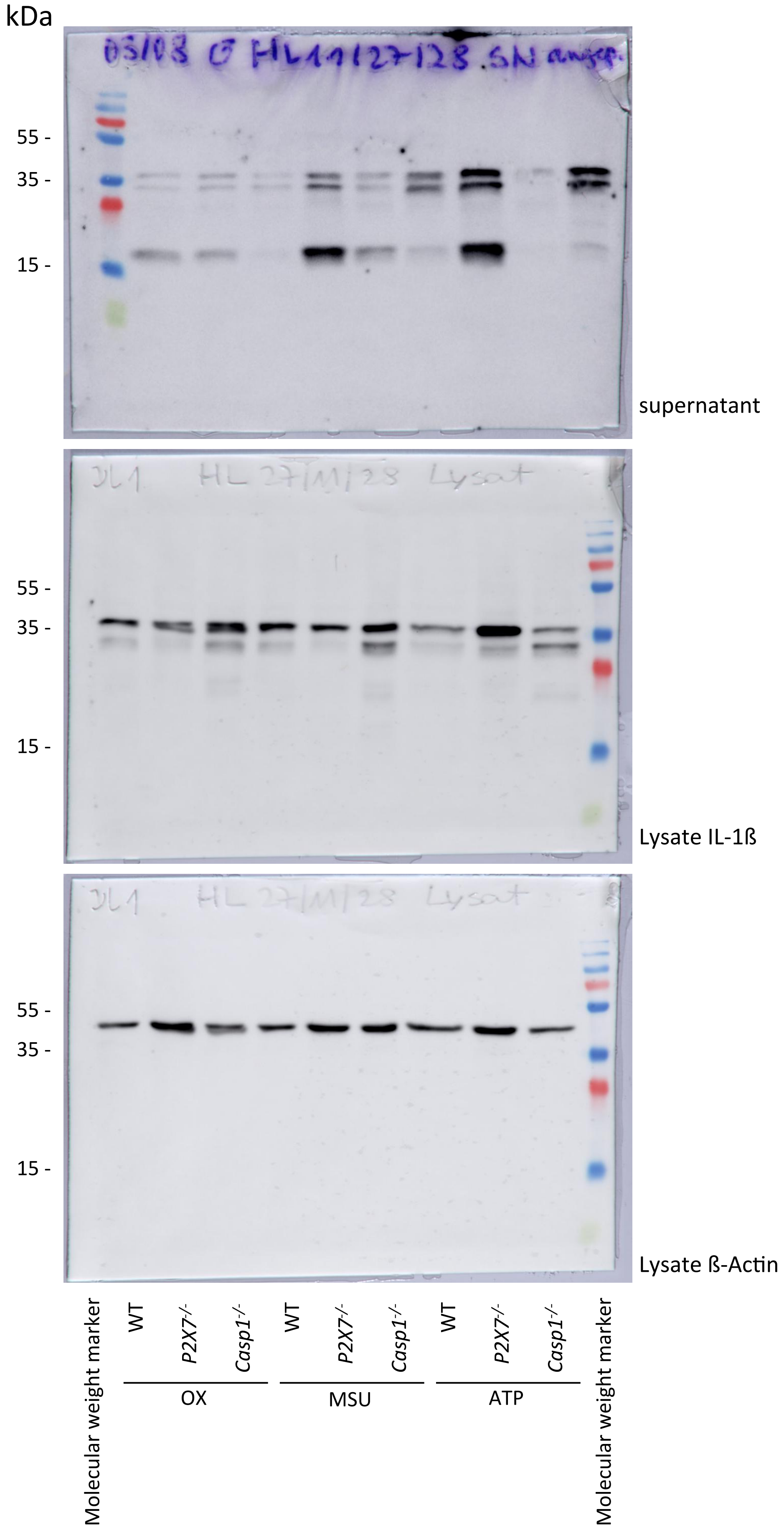

4 – qPCR confirming absence of P2X7 mRNA in P2X7$^{-/-}$ mice.

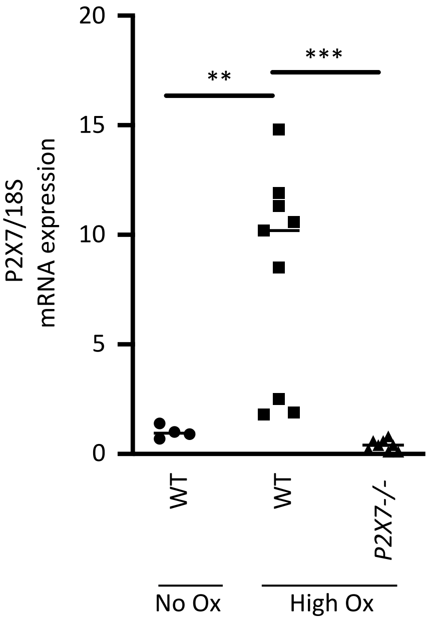

Data are presented as mean. n=4 for WT animals on control diet, n=9 for littermate WT on high soluble oxalate diet, n=8 for P2X7$^{-/-}$ animals. Statistical analysis was performed using one-way ANOVA. ***P<0,001; **P<0,01.

5 – Plasma BUN and creatinine in WT littermates and P2X7$^{-/-}$ mice *in vivo.*

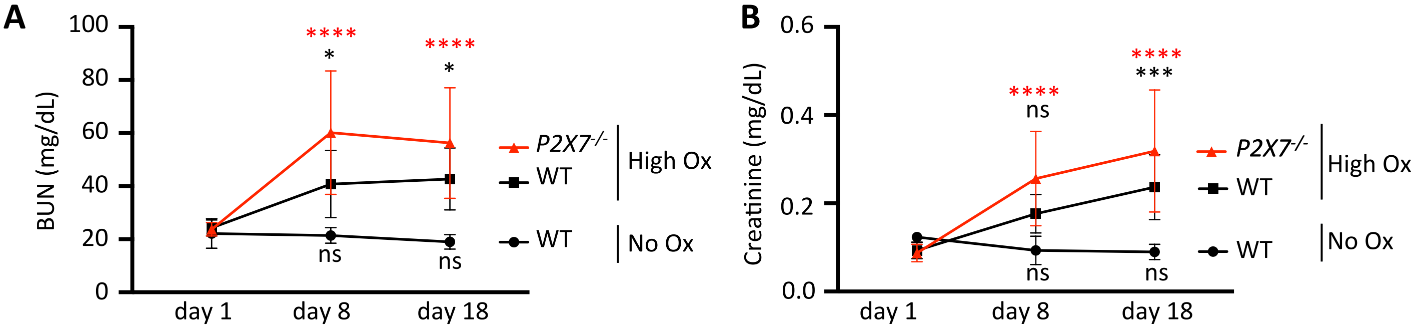

Data are presented as mean ± SD. n=4 for WT animals on control diet, n=9 for littermate WT on high soluble oxalate diet, n=8 for P2X7$^{-/-}$ animals. Statistical analysis was performed using two-way ANOVA. ****P<0,0001; ***P<0,001; *P<0,05; ns, not significant.

6 – P2X7$^{-/-}$ and WT littermate mice with oxalate-induced kidney injury demonstrate no difference in renal injury and fibrosis.

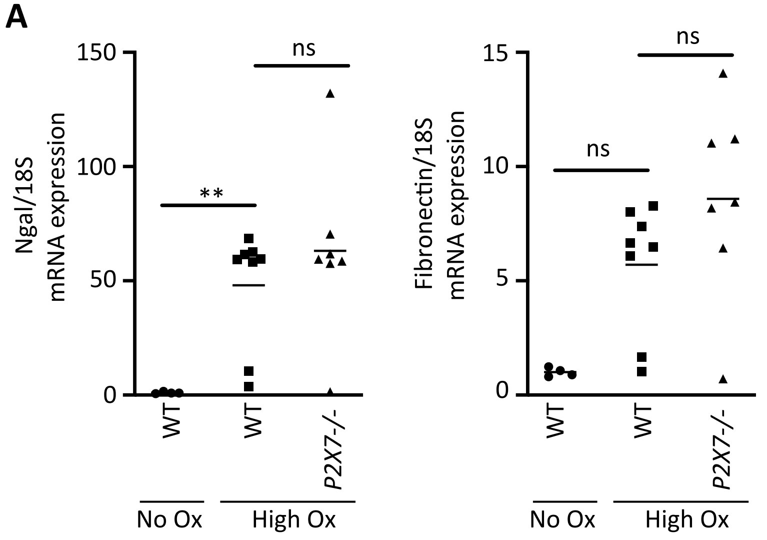

Data are presented as mean. n=4 for WT animals on control diet, n=8 for littermate WT on high soluble oxalate diet, n=8 for P2X7$^{-/-}$ animals. Statistical analysis was performed using one-way ANOVA. **P<0.01; ns, not significant.
